# Myometrial oxidative stress drives MED12 mutations in leiomyoma

**DOI:** 10.1186/s13578-022-00852-0

**Published:** 2022-07-22

**Authors:** Yinuo Li, Xiuhua Xu, Huma Asif, Yue Feng, Brendan F. Kohrn, Scott R. Kennedy, J. Julie Kim, Jian-Jun Wei

**Affiliations:** 1 Department of Pathology, Feinberg School of Medicine, Northwestern University, 251 East Huron Street, Feinberg 7-334, Chicago, IL 60611 USA; 2 Department of Obstetrics and Gynecology, Feinberg School of Medicine, Northwestern University, 303 E. Superior Street, 4-117, Chicago, IL 60611 USA; 3 Department of Laboratory Medicine & Pathology, University of Washington, Seattle, USA; 4 Lurie Cancer Center, Northwestern University, Chicago, IL USA; 5 Center for Reproductive Science, Northwestern University, Chicago, IL USA

**Keywords:** Leiomyoma, *MED12* mutation, Myometrium, Reactive oxidative species (ROS), 8-OHdG, Duplex sequencing

## Abstract

**Background:**

More than 70% of leiomyomas (LM) harbor *MED12* mutations, primarily in exon 2 at c.130-131(GG). The cause of *MED12* mutations in myometrial cells remains largely unknown. We hypothesized that increased ROS promotes *MED12* mutations in myometrial cells through the oxidation of guanine nucleotides followed by misrepair.

**Methods:**

Genomic oxidative burden (8-OHdG) was evaluated in vitro and in vivo by immunohistochemistry. *MED12* mutations were examined by Sanger sequencing and deep sequencing. Transcriptome examined by RNA-seq was performed in myometrium with and without LM, in primary myometrial cells treated with ROS. 8-OHdG mediated misrepair was analyzed by CRISPR/Cas9.

**Results:**

Uteri with high LM burden had a significantly higher rate of *MED12* mutations than uteri with low LM burden. Compelling data suggest that the uterus normally produces reactive oxidative species (ROS) in response to stress, and ROS levels in LM are elevated due to metabolic defects. We demonstrated that genomic oxidized guanine (8-OHdG) was found at a significantly higher level in the myometrium of uteri that had multiple LM compared to myometrium without LM. Transcriptome and pathway analyses detected ROS stress in myometrium with LM. Targeted replacement of guanine with 8-OHdG at *MED12* c.130 by CRISPR/Cas9 significantly increased the misrepair of G>T. Exposure of primary myometrial cells to oxidative stress *in vitro* increased misrepair/mutations as detected by duplex sequencing.

**Conclusions:**

Together, our data identified a clear connection between increased myometrial oxidative stress and a high rate of *MED12* mutations that may underlie the risk of LM development and severity in women of reproductive age.

**Graphical Abstract:**

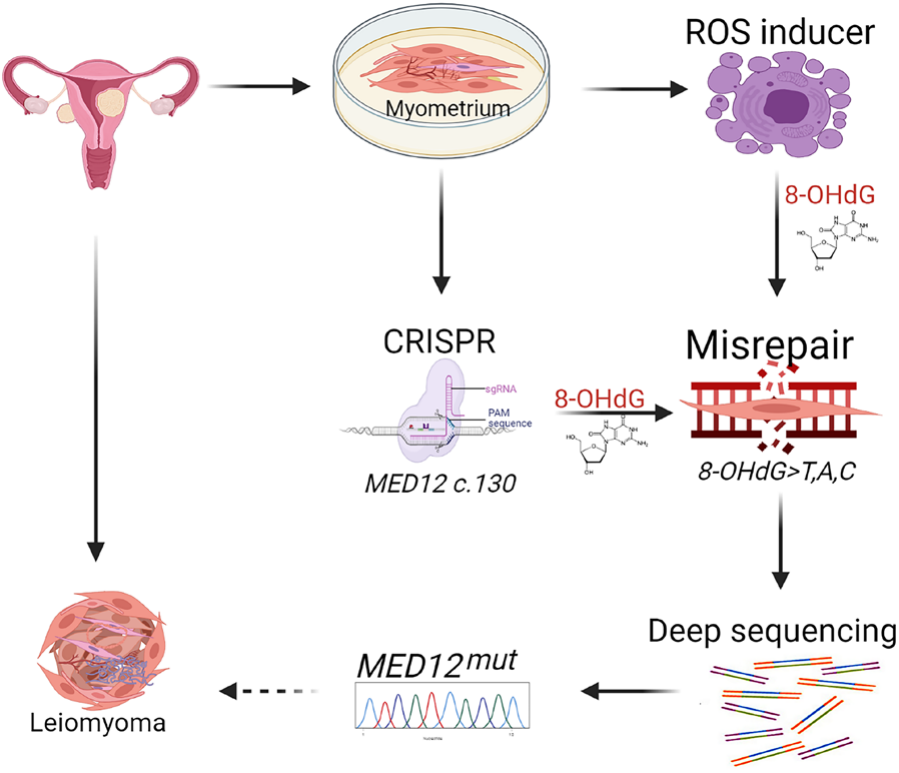

**Supplementary Information:**

The online version contains supplementary material available at 10.1186/s13578-022-00852-0.

## Highlights

High rate of *MED12* mutations and leiomyoma burden are clearly associated with myometrial oxidative stress.

*MED12* mutations are driven by misrepair of 8-OHdG *at* c.130-c.131 detected by deep sequencing analysis both in vitro and in vivo.

**Supplementary Information:**

The online version contains supplementary material available at 10.1186/s13578-022-00852-0.

## Background

Uterine leiomyomas (LM) occur in up to 77% of women of reproductive age and are the leading cause of approximately 600,000 hysterectomies/myomectomies per year in the U.S. [1, 2]. Health-related costs are estimated at $5.9 to $34.4 billion per year [3]. LM can cause significant morbidity, including profuse menstrual bleeding and pelvic pain as well as reproductive dysfunction. Whole genome sequencing studies have identified *MED12* mutations to occur in up to 70% of all LM [4, 5]. MED12 encodes a subunit of the mediator complex, which consists of at least 26 subunits and regulates transcription initiation and elongation by bridging regulatory elements in gene promoters to the RNA polymerase II initiation complex[4]. MED12 is essential for activating CDK8 and modulates mediator-polymerase II interactions for transcription initiation [6]. *MED12* mutations in uterine leiomyomas were mostly identified in exon 2 and rarely in intron 1-exon 2 junction.[4, 7] All mutations were heterozygous in genomic DNA, and all of the transcripts were derived solely from the mutant *MED12* alleles, suggesting its tumorigenesis role for LM development [8]. The molecular cause of these mutations remains unknown.

The uterus functions under conditions of high oxidative stress with elevated reactive oxidative species (ROS), in part, due to dynamic steroid hormone changes, homeostatic mechanisms, and ROS-related cell function [9–12]. Furthermore, studies have found a higher ROS burden in LM, related to a decreased ability to detoxify superoxides by protein acetylation of manganese superoxide dismutase at lysine 122 (MnSOD^K122Ac^) [12], and NADPH oxidase 4 (NOX4) dysfunction [13]. Although the ability of ROS to cause DNA mutations has been shown [14, 15], its effects on uterine myometrium are unknown. One common mutagenesis mechanism in response to ROS exposure is the accumulation of oxidative nucleotides in genomic DNA [16, 17], in particular, modified guanine 8-OHdG ((8-hydroxy-2'–deoxyguanosine). Through oxidization of the G nucleotide and subsequent mismatch pairing, ROS exposure can cause specific types of DNA mutations [18]. Although emerging data show that oxidized nucleotides in DNA can be readily fixed by base excision repair (BER) [19], high ROS levels continue to increase the misrepair rate of 8-OHdG and other modified nucleotides, which is a hallmark of ROS-mediated tumorigenesis [20]. 8-OHdG–mediated gene mutations have been reported for *K-Ras* and *p53* [14, 15], where a disproportionately high rate of missense mutations are seen in guanine transversions/transitions in *p53* at codons 175/248/273 [15] and in *K-Ras* at codon 12, particularly in lung cancer associated with tobacco smoking [15].

In this study, we test our hypothesis that ROS can promote mutations in the *MED12* gene through the oxidation of guanine nucleotides in human myometrial cells. We demonstrate that increased ROS is evident in the myometrium of uteri with high number of LM tumors, that treatment of myometrial cells *in vitro* with ROS inducers causes increased 8-OHdG, that insertion of oxidized guanines at c.130-131of *MED12* using CRISPR/Cas9 technology promotes misrepair and mutations, and that both acute and chronic treatments of myometrial cells promote mutations of *MED12* at sites similar to hot spot mutations found in LM tumors. Altogether, our study demonstrates for the first time, a clear connection between increased myometrial ROS and *MED12* mutations that may underlie leiomyoma development.

## Materials and methods

### Patient specimens

Uterine tissues were collected from premenopausal women undergoing hysterectomy or myomectomy at Northwestern University Prentice Women’s Hospital (Chicago, Illinois) from 2010 to2021. Tissues collected from a total of 309 women were included in this study. A total of 529 leiomyomas (LM) were subjected to *MED12* mutation analysis. In addition, 35 uteri without LM from age-matched hysterectomy (uterine bleeding, prolapse, and Adenomyosis) were included as controls. Pathology reports were reviewed and all LM were usual type. Patient age at surgery, race, uterine weight, number of tumors, tumor size, hormonal status, and hormonal treatment were documented. All LM and matched myometrium (MM) used for primary cell culture were from patients who had not received hormonal treatment. General information about the patient cohort is summarized in Additional file 2: Table S1**.**

### Cell culture and treatment

Myo-hTERT cell line was kindly provided by C. Mendelson (UT Southwestern) and cultured in DMEM/F12 medium (Thermo Fisher Scientific, Cat# 41966052) plus 10% Fetal Bovine Serum (FBS, Fisher Scientific). HUtSMCs were obtained from PromoCell (C-12575) and grown in Smooth Muscle Cell Growth Medium 2 (PromoCell, C-22062). Primary LM and matched myometrial cells were isolated as previously described [21]. All the cells were grown at 37 °C in a humidified cell culture incubator containing 5% CO_2_. Oxidative stress conditions were mimicked *in vitro* by the exogenous addition of Paraquat dichloride hydrate (PQ; Sigma Aldrich, 36541). An optimal dose for PQ (100 μM) was selected based on ~50% cell death after 48 h of treatment in a dose-response study. For acute treatment strategy, cells were treated with PQ for 24 h or 48 h; for chronic treatment, the cells were treated with PQ for 48 hrs and recovered in fresh media for 24 h. This process was then repeated 5–6 times.

### Myometrial spheroid/three-dimensional (3D) culture

Myometrial spheroids were cultured as previously described [21, 22]. Briefly, cells were plated in 96-well ultra-low attachment plates (Corning Costar), cultured in mesenchymal stem cell medium (Lonza, PT-3001), and incubated at 37 °C in a humidified incubator with 5% CO_2_ for at least 48 h. Spheroids were formed and evaluated under an inverted microscope.

### DNA extraction and Sanger sequencing

DNA extraction from fresh/frozen or FFPE tissues of tissue bank was performed using the ZYMO RESEARCH Quick-DNA™ MiniPrep Plus Kit and ZYMO RESEARCH Quick-DNA™ FFPE Kit (ZYMO RESEARCH, D4068, D3067, USA), respectively, according to the manufacturer’s protocol. 50 ng of genomic DNA was used to amplify *MED12* exon 2 for Sanger sequencing (primer see Additional file 2: Table S2). Sanger DNA sequencing of the purified DNA products was performed in NUSeq Core (Northwestern) with Applied Biosystem's 3730xl DNA Analyzer. Mutations/variations were analyzed by FinchTV and Indigo software (https://www.gear-genomics.com/indigo/).

### AP assay

Apurinic or apyrimidinic (AP or abasic) sites produced by ROS inducers are detected by AP Sites Quantitation Kit (Cell Biolabs) according to the manufacturer protocol. Genomic DNA (gDNA) was isolated from the cells treated with or without ROS inducers using DNAzol reagent and dissolved in TE buffer. gDNA was mixed with the aldehyde reactive probe (ARP) ) to react specifically with an aldehyde group on the open-ring form of AP sites. AP sites were tagged with biotin and detected with a streptavidin–enzyme conjugate. The quantity of AP sites per 10^5^ nucleotides was determined at 450 nm with a standard curve produced using ARP-DNA standard solutions.

### RNA extraction and RT-PCR

Treated primary cells were isolated with TRIZOL reagents (Invitrogen, Carlsbad, CA) according to the manufacturer's protocol. RNA was quantified with the NanoDrop (ND-1000, Saarbrücken), and cDNA was synthesized from 1 μg of total RNA using qScript™ cDNA Synthesis Kit (QuantaBio). Quantitative RT-PCR was performed using Power SYBR™ Green PCR Master Mix in an Applied Biosystems 7900HT Real-Time PCR System. Primer sequences are shown in Table S. Fold change values were calculated using the comparative Ct method using endogenous control GAPDH. The experiments were repeated in triplicate.

### Tissue microarray (TMA)

After slide review, the FFPE tissue of LM and matched myometrium were selected. Tissue cores of 1.5 mm in diameter were taken to create TMAs. TMAs were sectioned at 4 μm. The first and last slides of each TMA were stained with hematoxylin and eosin (H&E) for quality assurance to confirm the correct tissue types.

### Immunohistochemistry

Immunohistochemical staining was performed on a Ventana Nexus automated system at Northwestern Pathology Core Facility as described previously [5]. Antibody information is summarized in Additional file 2: Table S3, including antibodies for 8-OHdG and γH2AX. Immunostaining was scored semi-quantitatively by percentage and intensity by pathologists. The intensity and the percentage were combined as H-Score.

### Immunofluorescence and Dihydroethidium (DHE) Stain

The cell and tissue sections were washed with cold PBS and immediately fixed with cold methanol (−20°C) for 10 min, followed by incubation in 3% bovine serum albumin in PBS for 1 hr to block the nonspecific binding sites. A primary antibody to 8-OHdG (Santa Cruz) or γH2AX (Novus Biologicals) was added overnight at 4°C. After washing 3 times with 1X PBST, secondary antibodies and Alexa Fluor® 488-conjugated goat anti-mouse (1:1000, Life Technologies) were added for 1 hr at room temperature (Additional file 2: Table S3). Slides were counterstained with 4′,6-diamidino-2-phenylindole (DAPI) for analysis. For the DHE staining, the slides were incubated with the 5 μM DHE for 10 to 15 minutes in a dark chamber, on an orbital shaker at room temperature. After washing 3 times for 5 minutes/wash with 1X PBS and slight fixation for 4 to 8 minutes in 7% formaldehyde in 1X PBS, the slides were counterstained with DAPI, and images were captured using a fluorescence microscope.

### CRISPR/Cas9 and target sequence

c.129-131 (AGG) from *MED12* exon2 was selected as the Protospacer Adjacent Motif (PAM) site. The target-specific sequence for guiding Cas9 protein was: ACGGCCTTGAATGTAAAACA, and was inserted into Alt-R^®^ CRISPR/Cas9 crRNA (provided by Integrated DNA Technologies [IDT]). The template sequence for the ssDNA HDR donor (ACGCCGCATTCCTGCCTCAGGA TGAACTGACGGCCTTGAATGTAAAACAA/**i8-oxodG**/GTTTCAACAACCAGCCTGCTGT CTCTGGGGATGAGCATGGCAGTGCCAA) was designed and synthesized by IDT (Additional file 2: Table S2). **c.138T>C** was taken as the index in the genomic DNA. The electroporation of CRISPR/Cas9 was done using the Neon^®^ Transfection System Kit. In brief, crRNA and ATTO 550-tracrRNA were heated at 95 °C for 5 min to form the gRNA complex at a final concentration of 50 μM. Then, the gRNA complex (final concentration: 4.8 μM) and Alt-R Cas9 enzyme (final concentration: 4 μM) were mixed and incubated at room temperature for 10–20 min to form the RNP complex. 1 μl of 50 μM ssDNA template was added to 2.5 μl RNP complex and then the Cas9‐RNP+ssDNA mixture was prepared. 9 μl (10x10^6^ primary myometrial cells/ml) was resuspended in 9 μl buffer R in the PCR tube and 3.5 μl Cas9 RNP+ssODN was added. 10 μl tips were used for the electroporation at 1500V, 20ms with 2 pulses. The electroporated myometrial cells were plated into 6-well plates and then cultured for 18 h. The cell condition and ATTO 550 were checked using fluorescence microscopy. Cells were maintained in a regular medium for 5 days, then harvested for DNA extraction. The target sequences in treated cells were analyzed by deep sequencing (see below).

### Deep sequencing and duplex sequencing

To detect DNA misrepair/mutations in *MED12* exon2, genomic DNA was extracted and *MED12* exon 2 DNA was amplified for conventional deep sequencing or were captured for duplex sequencing analysis. For conventional deep sequencing, *MED12* exon 2 was amplified, followed by library preparation using the QIAseq 1-Step Amplicon Library Kit (Qiagen) according to the manufacturer’s instructions. The libraries were pooled and sequenced on an Illumina HiSeq4000 at NUseq Core, and sequencing reads were aligned to hg19 and assembled using Samtools-Mpileup and GATK4. *MED12* mutation frequency of each nucleotide of exon 2 was calculated as the number of reads with mutated allele divided by the total number of reads.

A more sensitive sequencing strategy, duplex sequencing detects very low rates of double-strand nucleotide alterations/mutations [23, 24]. For duplex sequencing, DNA was sonicated, end-repaired, A-tailed, and ligated with UMI (IDT) and index adapters (IDT) using the KAPA HyperPrep library kit (Roche Sequencing). After initial amplification, 120 bp biotinylated oligonucleotide probes (IDT) were used to capture the exon 2 DNA of *MED12* using xGen^®^ Lockdown^®^ Reagents (IDT) according to the manufacturer’s protocol. Two successive rounds of captures were performed to ensure sufficient target enrichment. The libraries were sequenced using 2 x 150 paired-end reads with eight-base indexing read on an Illumina NovaSeq 6000 at NUseq Core. Duplex sequencing data were processed using the previously published method [24]. Briefly, FASTQ files were converted to unaligned bam using Picard FastqToSam. Reads were grouped based on the unique tagged sequences to generate read families needed to make single-strand consensus sequences (SSCS) and duplex consensus sequences (DCS) using Unified consensus maker (UCM). Consensus sequences were aligned to the reference hg38 genome using BurrowsWheeler aligner (BWA-mem) and variants calling was performed using VarDict (Java). Later, for each mutation, mutation frequency was calculated as the number of DCS reads showing the mutant allele divided by the total number of DCS reads. All the sequencing data were summarized in Additional file 2: Table S4.

### RNA sequencing

Total RNA was isolated using the Qiagen miRNeasy Mini Kit according to the manufacturer’s instructions (Qiagen, cat. # 217004). These included fresh frozen tissue of MM with and without LM (n=3 each), 4 groups of cultured myometrial cells (controls and PQ treatment, n=4 each). RNA samples were quantified using a Qubit 2.0 Fluorometer (Life Technologies) and RNA integrity was checked using Agilent TapeStation 4200 (Agilent Technologies). RNA integrity numbers (RIN) greater than or equal to 9 were used for library preparation using the NEBNext Ultra II RNA Library Prep Kit for Illumina following the manufacturer’s instructions (New England Biolabs/NEB, Ipswich, MA). Briefly, mRNAs enriched using Oligo (dT) beads were fragmented for 15 minutes at 94 °C. First-strand and second-strand cDNAs were then synthesized. cDNA fragments were end-repaired and adenylated at the 3’ ends, and universal adapters were ligated to cDNA fragments, followed by index addition and library enrichment by limited-cycle PCR. The sequencing libraries were validated on the Agilent TapeStation (Agilent Technologies) and quantified using a Qubit 2.0 Fluorometer (Invitrogen), as well as by quantitative PCR (KAPA Biosystems). RNA sequencing was performed by Genewiz (Genewiz, NJ) on the Illumina HiSeq4000 and sequenced using a 2x150bp Paired End (PE) configuration.

### RNA-seq data processing and analysis

The quality of DNA reads, in FASTQ format, was evaluated using FastQC (Babraham Bioinformatics). Adapters were trimmed and reads of poor quality or aligned to rRNA sequences were filtered out. The clean reads were mapped to the Homo sapiens GRCh38 reference genome available on ENSEMBL using the STAR aligner v.2.5.2b, and featureCounts from Subread package v.1.5.2 was applied to calculate unique gene hit counts. After extraction, the gene hit counts table was used for downstream differential expression analysis using the DESeq2 package in R. Differentially expressed genes were identified using threshold adjusted p-value < 0.05 and absolute log2 fold change >0.5 (for Control vs. PQ). To perform clustering analyses on a group of samples, a union of all the genes and their expression RPKM values within that group was generated to build a read count matrix for the group of interest. Various unsupervised cluster analysis and other machine learning techniques were applied to the composite read count matrix of interest. The packages ggplot2 and Pheatmap in R were used to build various heatmaps and volcano plots. “Factoextra and FactoMineR” packages were used for principal component analysis (PCA) to reveal the similarity between samples based on the distance matrix. The “fgsea” and “enrichplot” packages were run for gene ontology (GO) and Kyoto Encyclopedia of Genes and Genomes (KEGG) analysis. Functional analysis was performed by Gene Set Enrichment Analysis (GSEA) using the Molecular Signatures Database, MSigDB (http://www.broad.mit.edu/gsea/msigdb/index.jsp). RNA sequence data have been submitted to GeneBank (SRA accession number: PRJNA746002) and the significantly changed genes were summarized in Additional file 2: Table S5.

### Statistical analysis

Statistical analysis of RNA-Seq was performed using related packages in R. A two-way ANOVA (paired *t*-test) was constructed to identify genes that were differentially expressed in all LM compared with the corresponding myometrium samples. A one-way ANOVA was constructed to identify genes that were differentially expressed between each LM subtype and the myometrium samples. Another statistical analysis was performed using GraphPad Prism version 8.0 (GraphPad Software). Unpaired t-tests were performed when comparing two groups. Paired t-test was used when comparing the two treatments. Ordinary one-way ANOVA, Brown-Forsythe and Welch ANOVA, or Kruskal-Wallis test was performed for multiple comparisons depending on the distribution and variances of the data. All data represent the mean ± SEM of a minimum of three independent experiments and data were considered statistically significant if the p value was < 0.05.

### Study approval

The study protocol for tissue donation was approved by Northwestern Institute Review Board (IRB) and informed consents were obtained from all patients following an IRB-approved protocol.

## Results

### MED12 mutations are associated with LM burden

**Fig. 1:**
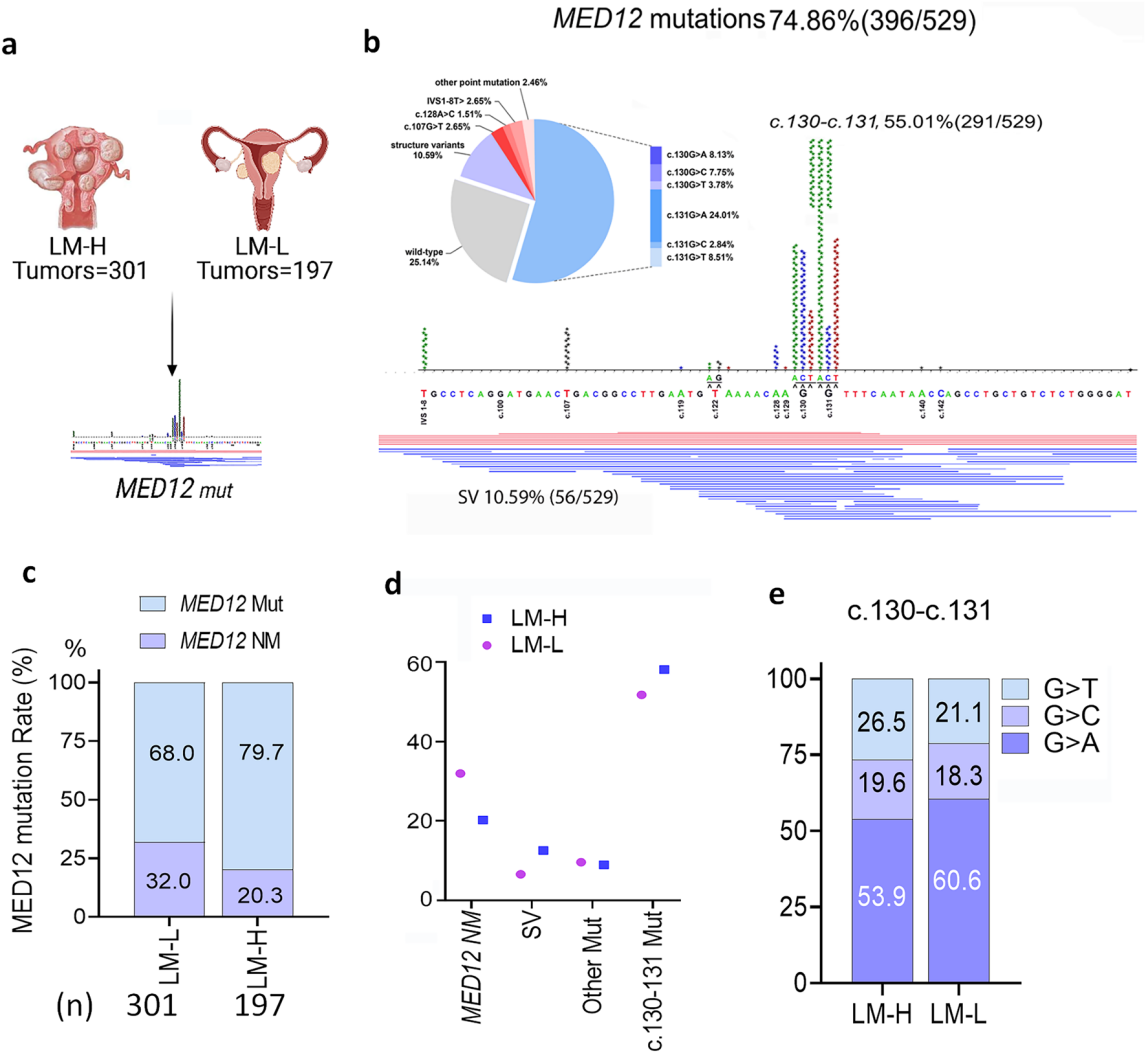
*MED12* mutation analysis in LM based on number of LM/uterus. **a** A diagram illustrated the number of patients and LM collected from the number of tumors (LM-H: ≥5 LM/uterus; LM-L: <5 leiomyoma/uterus). **b** *MED12* mutation patterns and distribution in 529 LM. Pie chart illustrated the percentage of each mutation type. The actual mutation distribution in *MED12 exon 2* and intron 1-exon 2 boundary were illustrated along with a sequence map. Each dot represents one mutation. Blue lines under the sequence indicated the actual deletion site and extension below the graph. Red lines indicate complex structure changes in exon 2. *MED12* Mut: Leiomyomas with *MED12* mutation; *MED12* NM: Leiomyomas without *MED12* mutation. **c** Percentage of *MED12* mutations (light blue) in LM-H and LM-L uteri. The number of tumors (n) were shown and number of LM per uterus (LM-H: blue; LM-L: purple). **d** Differences in *MED12* mutation types were NM: non-mutation; SV: Structure variants, insertions/deletions; other mut: all point mutation other than c.130-131; c.130-131 mut: mutation at codon44 c.130 and c.131 guanine. **e** Percentage of c.130-131 missense mutations G>A (dark blue), G>C (medium blue), and G>T (light blue) by number of LM per uterus

Mutations in the *MED12* gene are prevalent in uterine LM and thus have been implicated in LM tumor development [4]. In order to determine whether *MED12* mutation rate is affected by the LM burden of the uterus, the incidence of *MED12* mutations was analyzed on a large cohort of 529 tumors (Fig. 1a). Among them, 301 LM were from uteri with more than 5 LM, which we call LM-high (LM-H), 197 were from uteri with 1-4 leiomyomas, called LM-low (LM-L) and 31 were from uteri with an unknown number of LM/uterus (Fig. 1a, Additional file 2: Table S1). We observed that the *MED12* mutations were highly enriched at c.130-131 (55.0%)*,* followed by deletions (10.6%) and other point mutations at eight other sites (9.3%; Fig. 1b). A significantly increased *MED12* mutation rate was seen in LM-H compared to LM-L (79.7 % vs. 68.0%; p<0.01; Fig. 1c). Given the noticeable difference in *MED12* mutations between LM-H and LM-L, we performed a comparative analysis of all mutation types including point mutations at c.130-131, other point mutations, and insertion/deletions, and observed that point mutations at c.130-131 and insertion/deletions were higher in LM-H compared to LM-L (p<0.01; Fig. 1d). When comparing the types of missense mutations at c.130-131, G>A (53.9% in LM-H, 60.6% in LM-L), followed by G>T, and G>C, no significant differences were noted between tumor number groups (Fig. 1e). *MED12* mutation patterns and distributions in association with tumor number are summarized in Additional file 1: Fig. S1a-1b. These data demonstrate that increased LM burden is associated with higher *MED12* mutation rate, implicating the importance of the microenvironment that promotes *MED12* mutations.

### Transcriptome analysis reveals myometrium with LM has a high ROS burden and oxidative stress response

**Fig. 2:**
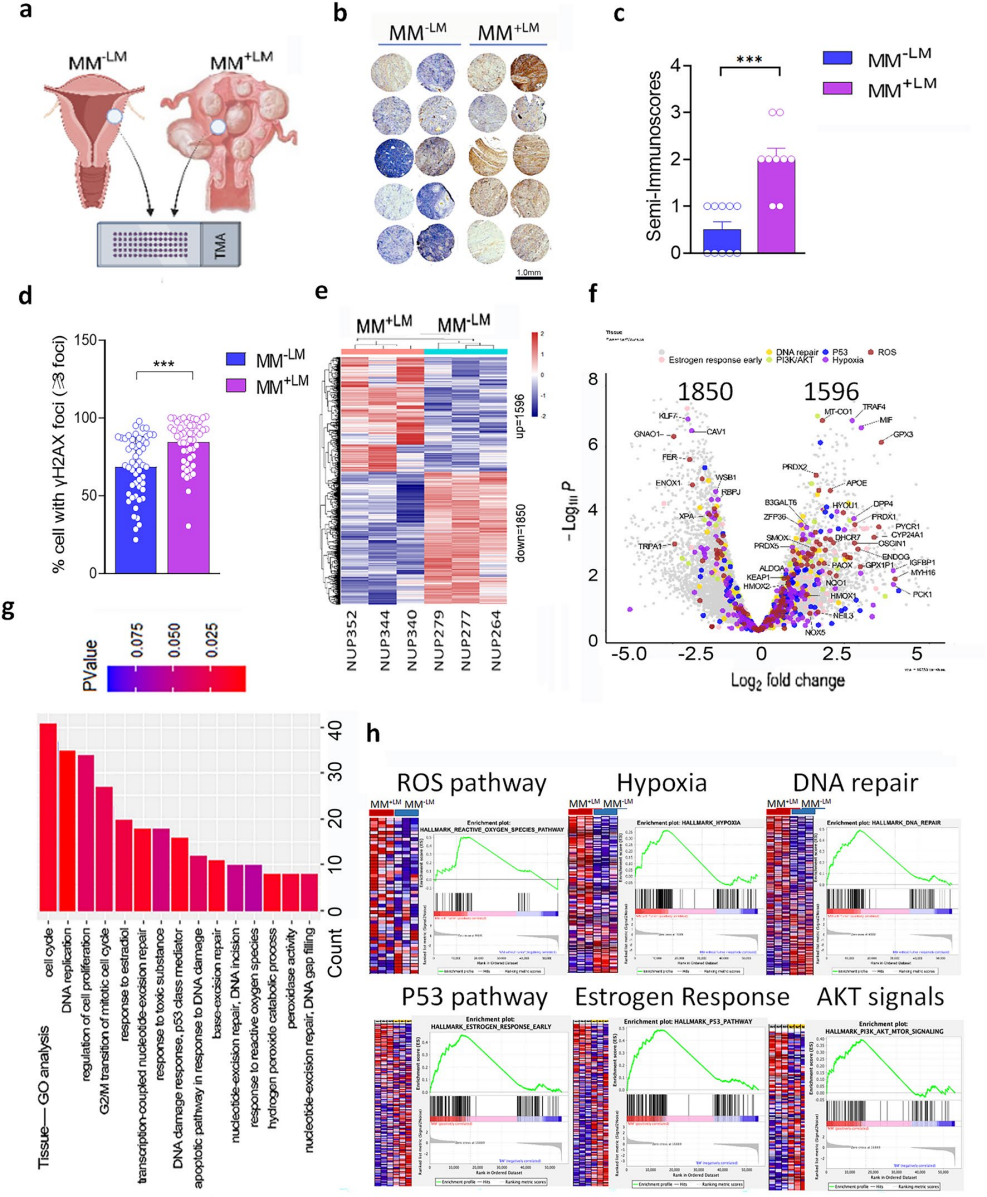
Oxidized DNA, DNA damage response, and ROS response in myometrium with or without LM.** a** Diagram illustrated tissue microarray (TMA) preparation in myometrium without LM (MM^-LM^) and with LM (MM^+LM^). **b** Immunohistochemistry for 8-OHdG in MM^-LM^ and MM^+LM^. **c** Histobars showed semi-quantitative immunoscores for 8-OHdG in MM^-LM^ and MM^+LM^ (n=10 for each). **d** Percentage of cells with ≥3 nuclear dots of immunofluorescent stain for γH2AX in MM^-LM^ and MM^+LM^ (n=10 for each). **e** Heatmap showed the expression of 3446 genes were significantly different between MM^-LM^ and MM^+LM^ (n=3 for each). (MM^+LM^ vs. MM^-LM^: 1,596 upregulated and 1,850 downregulated). **f** Volcano plot compared a global transcriptional change across the groups (MM^-LM^ vs. MM^+LM^, n=3). Genes involving DNA repair (yellow), P53 (blue), ROS (red), AKT (green) and hypoxia (purple) pathways were highlighted. **g** GO pathway analysis revealed the top-ranking pathways different in MM^+LM^ in comparison to MM^-LM^ (p value ranked from low in red to high in blue). **h** Pathway analysis from GSEA revealed significantly changed pathways: ROS, hypoxia, oxidative phosphorylation and DNA repair pathways. *p<0.05; **p<0.01

The mechanisms associated with the development of LM tumors remain unknown. The increased *MED12* mutation when LM burden is high suggests that the signals from LM promote *MED12* mutations. Our previous studies demonstrated that LM are under high oxidative stress related to ROS metabolic defects [12, 21]. In order to evaluate the naturally occurring ROS burden in the myometrium, we examined a subset of age-matched myometrial tissues from women with LM (MM^+LM^) and without LM (MM^-LM^). As ROS is able to oxidize deoxyguanosine in DNA, 8-OHdG was used as a biomarker for oxidative stress. Immunohistochemistry (Fig. 2a) and immunofluorescence staining (Additional file 1: Fig. S2a) for 8-OHdG, were performed in MM^+LM^ or MM^-LM^ in a tissue microarray (TMA, Fig. 2a, b). Significantly higher immunoreactivity for 8-OHdG was observed in MM^+LM^ compared to MM^-LM^ (Fig. 2c; p<0.001), suggesting that MM^+LM^ have a higher ROS burden than MM^-LM^. As oxidized DNA 8-OHdG accumulation has been found to overlap with DNA damage [25], we examined γH2AX, DNA damage in the same samples by immunofluorescence staining (Fig. Additional file 1: Fig. S2b). Quantification of the γH2AX immunofluorescent signals was significantly higher in MM^+LM^ compared to MM^-LM^ (Fig. 2d).

To evaluate the gene expression differences in myometrium with and without LM, RNA sequencing analysis was performed in age-matched MM^+LM^ and MM^-LM^. Expression analysis revealed a total of 3446 genes were significantly different between MM^+LM^ and MM^-LM^ (1596 upregulated and 1850 downregulated with p-value < 0.05 and absolute log2 fold change > 1) (Fig. 2e, Additional file 2: Table S5). Volcano plots illustrated gene expression in fold change and level of significance and genes in ROS, DNA repair, p53, and hypoxia were highlighted and most of them were upregulated (Fig. 2f). Pathway analysis by Gene Ontology (GO) revealed cell proliferation and cell cycle, ROS pathways, DNA repair and DNA damage response pathways were strongly associated with MM^+LM^ (Fig. 2g). Gene Set Enrichment Analysis (GSEA) revealed that MM^+LM^ were significantly associated with ROS pathway, hypoxia, DNA repair, p53 pathway, estrogen response, and AKT signaling (Fig. 2h). Transcriptome and pathway analyses further defined defects in the uterine with LM in response to ROS stress.

### Stable ROS-mediated 8-OHdG, DNA damage response, and global gene expression in myometrium can be recapitulated in vitro

**Fig. 3:**
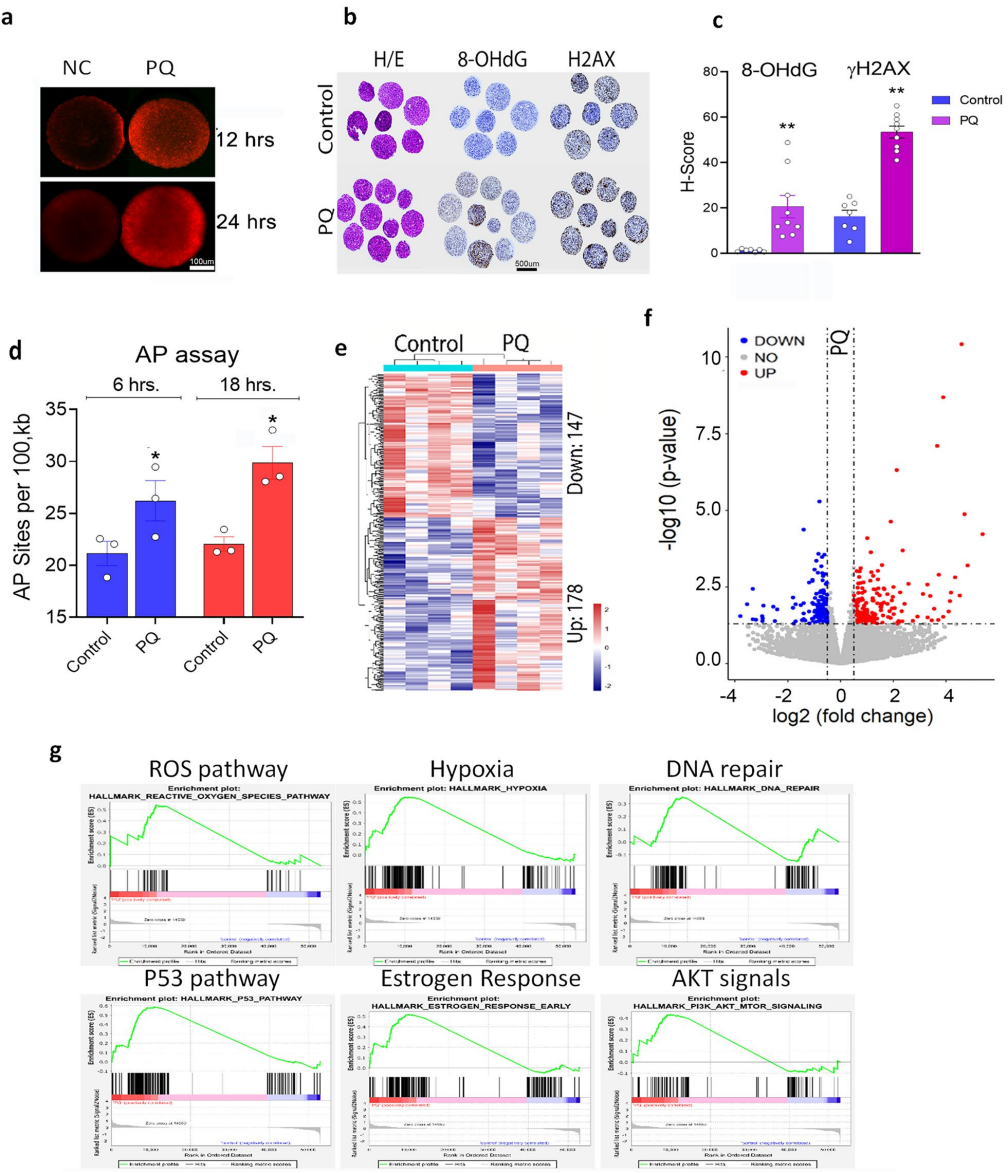
Evaluation of oxidized guanine in primary myometrial cells with ROS exposure *in vitro*, and ROS-induced gene expression in myometrial cells by RNA-seq analysis.** a** DHE staining (ROS) in 3D spheroid cultures of myometrial cells treated with control and PQ. **b** and **c** Immunohistochemistry (**b**) and quantitative H-scores (**c**) for 8-OHdG and γH2AX in 3D cultured myometrial cells treated with control and PQ. **d** Quantitative analysis of the absence of base site (apurinic/apyrimidinic (AP)) in genomic DNA treated for 6 hrs (blue bars) and 18 hrs (red bars) in myometrial cells treated with control and PQ . **e** Heatmaps (n=4, NUP245, NUP246, NUP237, NUP240) showed 325 genes treated with PQ (178 upregulated and 147 downregulated). **f** Volcano plot illustrated significantly up (Red) and down (blue) regulated genes. **g** Gene enrichment analysis of six major pathways upregulate by PQ treatment. *p<0.05; **p<0.01

To demonstrate that ROS directly promotes DNA oxidation and damage and changes genes expression*,* primary myometrial cells were treated with ROS inducer, Paraquat (PQ) [21], and responses were measured. Following dose-response studies, myometrial cells were treated with 100 µM PQ for 24 hours which showed the greatest oxidative stress with minimal cell death (Fig. 3a). Fluorescent staining was performed to detect ROS (dihydroethidium [DHE]), oxidized DNA (8-OHdG), and DNA damage (γH2AX) (Additional file 1: Fig. S3a-3b). Myometrial spheroids treated with PQ showed high ROS level (Fig. 3a) and histologic analysis showed no significant cytohistologic change or cell death on histology evaluation, however, a significant increase in immunoreactivity for 8-OHdG, and γH2AX were found in PQ-treated cells compared to untreated controls (Fig. 3b and c). Since apurinic/apyrimidinic (AP) sites are closely associated with 8-OHdG in genomic DNA mediated by OGG1, the primary enzyme responsible for the excision of 8-OHdG [26], we performed AP site analysis. As shown in Fig. 3d, primary myometrial cells treated with PQ resulted in a significantly higher number of AP sites in genomic DNA than controls. Together, these findings indicate that exposure of myometrial cells with ROS inducers causes DNA oxidation leading to 8-OHdG and DNA damage.

To evaluate the gene expression patterns in myometrial cells treated with PQ, RNA sequencing analysis was performed. After normalizing the data with an absolute log2 fold change cutoff of 1 (for Control vs. PQ), a total of 325 genes were dysregulated in cells treated with PQ (178 upregulated and 147 downregulated, Fig. 3e and f, Additional file 2: Table S5). GSEA revealed the dysregulated genes are significantly associated with the ROS pathway, hypoxia, DNA repair, p53 pathway, estrogen response, and AKT signaling (Fig. 3g), consistent with findings in myometrial tissue with LM (Fig. 2g and h), suggesting common mechanisms of the ROS-mediated DNA damage response to these two compounds. Upregulation of AKT pathways by PQ (Fig. 2h and 3g) was consistent with our previous functional analysis [21]. Genes in ROS stress response and the DNA repair pathway were mostly upregulated in PQ treatment (Additional file 1: Fig. S4a). The pathway connections and gene expression trends are summarized in Additional file 1: Fig. S4b. These findings demonstrate a common effect of PQ on ROS metabolic pathways, with DNA damage response, specifically the base excision repair pathway were dysregulated in PQ treatment.

### ***CRISPR/Cas9 editing of c.130***^***8-oxodG***^ results in DNA misrepair in myometrial cells

**Fig. 4:**
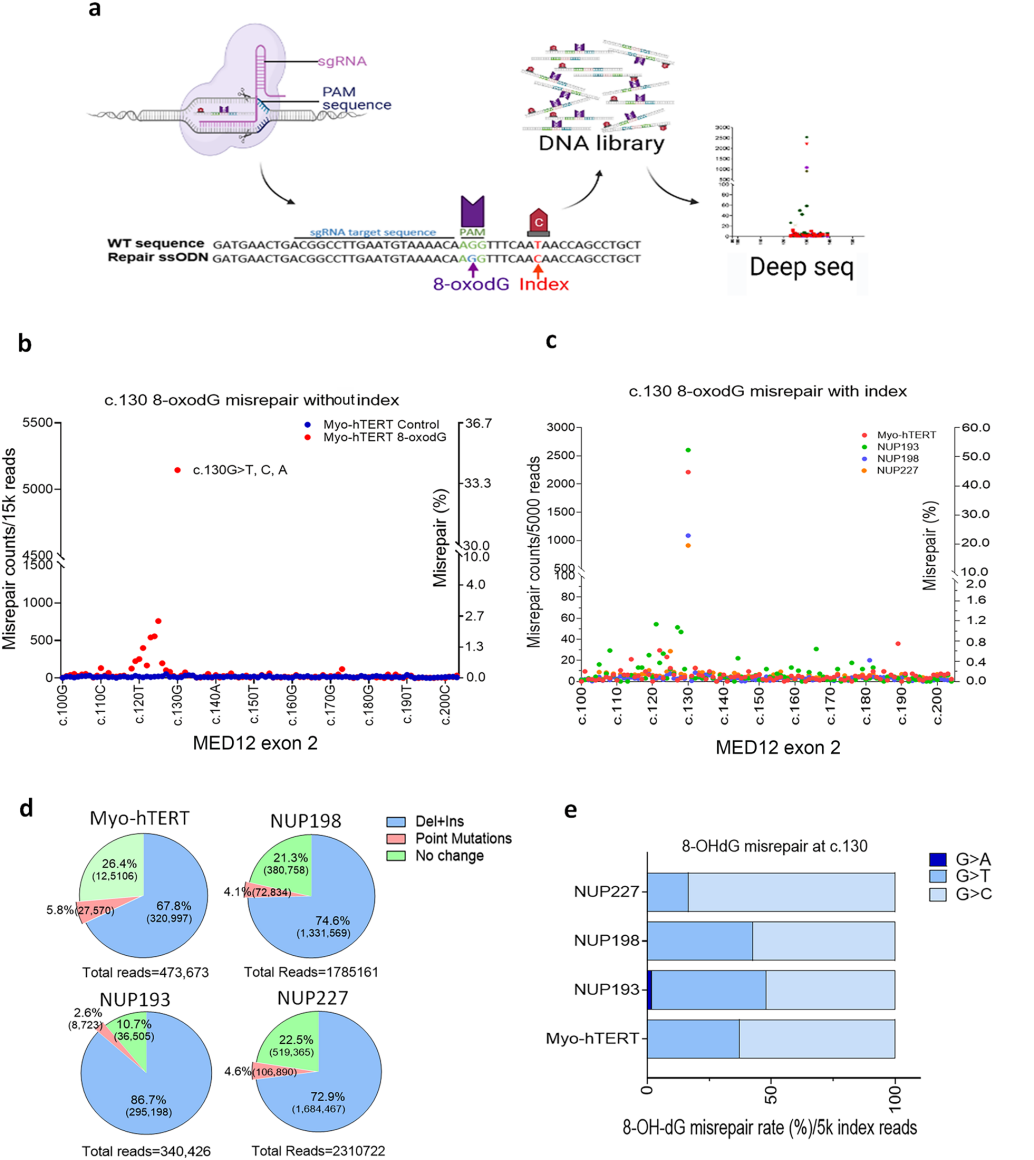
CRISPR/Cas 9-mediated targeted replacement of c.130G with 8-oxodG and misrepair analysis in myometrial cells. **a** A diagram illustrates the targeted replacement of c.130G with modified guanine (8-oxodG) on the ssDNA donor by CRISPR/Cas9, followed by deep sequencing analysis of misrepair caused by 8-oxodG. c.138T>C was used as an index. **b** Dot plot revealed the misrepair reads (c.130G>T, C, A: 34.3%) in the myometrial myo-hTERT cell line detected by lower depth (15k reads/sample) deep sequencing analysis after CRISPR/Cas9 editing to c.130^8-oxodG^ (red dots) and without 130G editing control (blue dots). **c ** Dot plot illustrated c.130G>T, A, C misrepair (44.2, 52.1, 21.7 and 18.2%, respectively) in the 4 myometrial samples with CRISPR/Cas9 editing to c.130^8-oxodG^ in *MED12* exon2 with the c.138T index in high depth deep sequencing analysis (500k reads/sample).  **d**. Pie graphs showed the percentage of different mutation types in myo-hTERT cells and three cases of primary myometrial cells with CRISPR/Cas9 editing. **e** Histobars showed the percentage of *c.130G>T, A, C* misrepair in four myometrial samples (n=4).

High ROS promotes DNA nucleotide oxidization, such as modified guanine 8-OHdG [25]. The 8-OHdG adduct, a typical product of oxidative DNA damage, can cause misrepair or mutation of guanine [18]. To demonstrate that oxidized guanine can indeed promote misrepair and mutations, CRISPR/Cas9 was used to replace guanine at codon44 c.130G of the *MED12* gene with an oxidized derivative of deoxyguanosine, 8-oxo-dG adduct (c.130^8-oxodG^). Technical details are summarized in Methods and Fig. 4a. c.130^8-oxodG^ and normal control were introduced into a myometrial cell line (myo-hTERT, Fig. 4b). c.130^8-oxodG^ mutations were detected by deep sequencing. Among a total of 15k reads, approximately 34.3% of c.130^8-oxodG^ showed misrepair of G>T, A, C, as demonstrated by the large peak at c.130 in the myometrial cell line (Fig. 4b, Additional file 2: Table S4). This data shows the extent of misrepair at this particular codon. In comparison, normal controls showed a baseline misrepair rate at c.130^G^. To accurately calculate the misrepair rates at c.130^8-oxodG^, an index nucleotide change at c.138^index^ (T>C, no amino acid change) along with c.130^8-oxodG^ from an ssDNA donor sequence was introduced into primary cultures of myometrial cells (n=3) and myo-hTERT cells. Misrepair at c.130^8-oxodG^ was examined by a high depth deep sequencing analysis (depth of 500k to 1 million reads /sample, Fig. 4c, Additional file 1: Fig. S5a). As shown in Fig. 4d (Additional file 2: Table S4), the c.130^8-oxodG^ misrepair rate in exon sequences with c.138^index^ was 16-46%. This is consistent with previous data from Manabu et al. [27] who used the TATAM (tracing DNA adducts in targeted mutagenesis) system to investigate the consequent mutations of synthetic 8-oxodG introduced into the human genome. The most common misrepair of c.130^8-oxodG^ was G>T, accounting for 96.0%-99.4% of mutations, whereas G>A represented a small fraction (0.3%-3.8%) of total misrepair and G>C accounted for 0.1–0.4% of misrepair (Fig. 4e). Furthermore, CRISPR/Cas9 targeted replacement of 8-oxodG at codon44 c.130 was reproducible in primary myometrial cells, but the success rate of site editing (based on presence of index c.138C) varied from case to case (ranging from 0.63 to 1.32%, Additional file 1: Fig. S5b), consistent with published data [28]. Of note, structural changes accounted for more than 75% of sequences (Fig. 4d, Additional file 1: Fig. S5b). Increased point mutations at c.123-126 AAAA might relate to the repeated adenosine sequence which is around the Cas9 cut site near the PAM site. (Fig. 4b and Additional file 1: Fig. S5a)*.* Taken together, this data demonstrates as proof of concept the significant consequences that the replacement of G with 8-OHdG has on misrepair and mutation.

### PQ treatment induces misrepair of MED12 exon 2 in myometrial cells in vitro

**Fig. 5:**
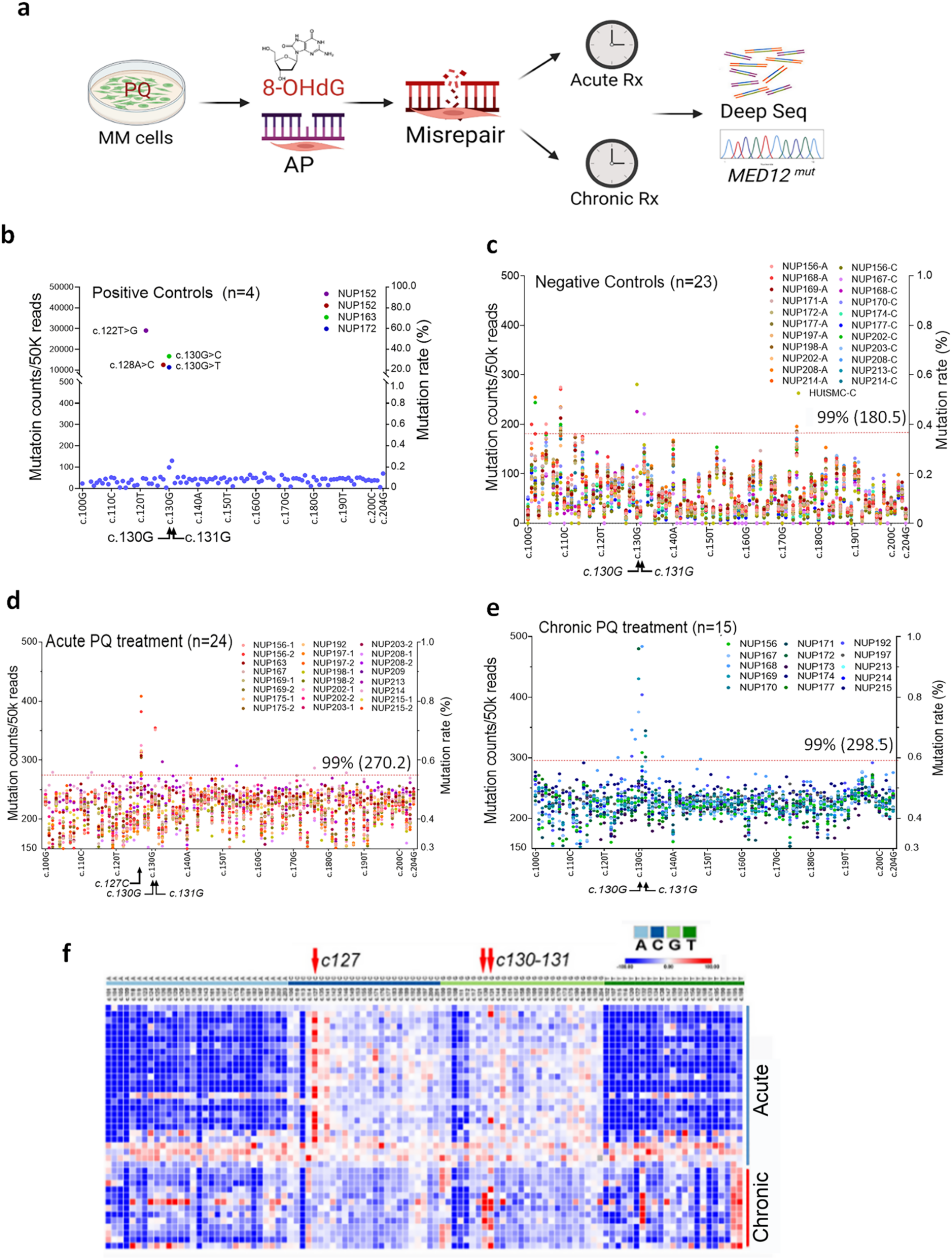
Deep sequencing analysis of *MED12* exon 2 mutations in primary myometrial cells with oxidative exposure *in vitro*.** a** Diagram illustrated the PQ treatment in primary myometrial cells, oxidation of DNA change, abasic (AP) DNA damage, and potential misrepair detected by deep sequencing for *MED12* exon 2 in a short- (acute) and long-term (chronic) PQ treatment. **b-e.** (**b**) Counts of *MED12* point mutations in four LM with known *MED12* mutations (positive controls) originally identified by Sanger sequencing (positive controls of c.122, c.128, c.130, and c.130 mutations with detected mutation rates of 58.1, 25.0, 33.3 and 22.7% in the number and distribution of *MED12* mutations in exon 2 counted by 50k reads/sample in deep tumor cells. (**c**) Counts of *MED12* point mutations in 23 primary culture samples of myometrial cells without treatment (11 for acute (A) and 12 chronic (C) treatment controls). (**d**) Counts of *MED12* point mutations in 24 primary culture samples of myometrial cells treated with PQ (100-200 μM) for up to 48 or 72 hrs (acute treatment). (**e**) Counts of *MED12* point mutations in 15 primary culture samples of myometrial cells treated with PQ (100 μM) for 5 times, with each treatment cycle maintained for up to 48 hrs (chronic treatment). **f** Heatmap illustrated the relative frequency (high in red, low in blue) of *MED12* exon2 mutations after acute (top) and chronic (bottom) PQ treatment. Mutation distribution of TCGA in each is marked on top. Red arrows highlight frequent mutations at c.127 and c.130-131

Thus far, we have demonstrated that the myometrium from a uterus with LM exhibited increased ROS, that ROS inducers cause oxidation of DNA, 8-OHdG, which can eventually be misrepaired and mutated as demonstrated using replacement of G with oxidized G at c.130 of the *MED12* gene. Next MM cells were exposed to either acute or chronic treatment with PQ (Fig. 5a) to determine the effects on exon2 of *MED12*. LM samples with known *MED12* mutations served as positive controls and showed *MED12* mutations at c.122, c.128, c.130, and c.130 were present at rates of 58.1, 25.0, 33.3, and 22.7%, respectively, based on a total of 50k reads/sample (n=4, Fig. 5b). Sequencing of MM samples treated with an acute or chronic dose of vehicle control (DMSO; Fig. 5c) showed a low mutation rate per nucleotide (acute: 0.12%; chronic: 0.10%) through the c.100-c.204 region (Fig. 5c).

Twenty-four myometrial samples were treated with PQ once (acute) and the DNA sequence of *MED12* exon 2 showed a higher rate of nucleotide alterations (average 0.42% per nucleotide) (Fig. 5d, Additional file 2: Table S4). Of note, c.127C, c.130G and c.131G alterations reached an average rate of 0.56%, 0.46% and 0.49%, respectively. Among these, 11 out of 24 myometrial samples harbored misrepair of c.127C, and 2 out of 24 harbored misrepair of c.130-131GG above cut-off (Fig. 5d). A total of 15 myometrial samples were treated with PQ five times (chronic). Deep sequencing analysis revealed that overall baseline mutations/alterations were similar to those seen with acute treatment, with no statistical difference (average 0.417% vs. 0.416%, p>0.05). To our surprise, 6 of 15 myometrial samples harbored c.130G,131G mutations above the cut-off line with an average mutation rate per nucleotide reaching 0.52% (c.130G) and 0.57% (c.131G), respectively. Three of 15 myometrial samples in this group showed a high mutation rate at c.132T above the cut-off (Fig. 5e, Additional file 2: Table S4). Interestingly, acute and chronic treatment resulted in different *MED12* mutation hotspots (Fig. 5f), suggesting a potential cell selection mechanism or non-random targeted DNA strand breaks and repair [29]. Next, we analyzed the ratio of G/C and T/A mutations and found that the G/C mutation rate is significantly higher than the A/T mutation rate (Fig. 5f). When all guanines were extracted from *MED12* exon 2 and the misrepair pattern of G>T, A, C examined, we found high G>A, followed by G>T, and G>C showed the lowest misrepair pattern (Additional file 1: Fig. S6a). These findings suggest that *in vitro* ROS exposure can increase mutations in *MED12* exon 2 and selected point mutations are non-randomly presented at unproportionally high rates above chance alone.

### Duplex Sequencing confirms high rate of MED12 mutations in myometria with oxidative burden both in vitro and in vivo

**Fig. 6:**
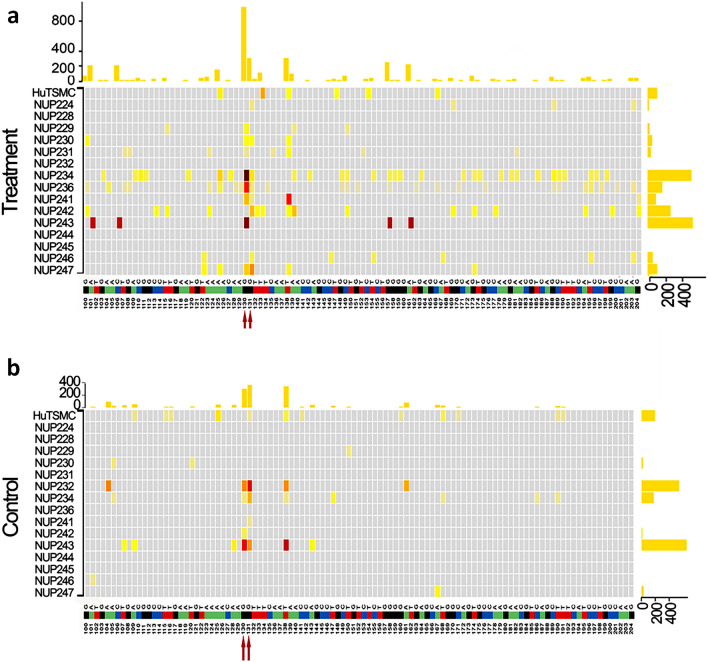
Duplex sequencing analysis of *MED12* exon 2 mutations in primary myometrial cells with PQ treatment and native myometrial tissue. **a**–**b**. Heatmap illustrated the accurate distribution of *MED12* mutation rates in exon 2 detected by duplex sequencing analysis in 16 myometrial samples with long-term PQ treatment (**a**) or with vehicle controls (**b**)

Given the error rate of conventional deep sequencing, we used duplex sequencing technology to detect bona-fide mutations with high accuracy and low rate of error by single strand-nucleotide change, effectively eliminating the chance of PCR errors (see Methods). A new cohort of 16 myometrial cases was exposed to chronic PQ treatment and duplex sequencing was performed. A similar mutation pattern in exon 2 with high misrepair/mutations at c.130-131GG was observed (Fig. 6, Additional file 2: Table S4). In the control group, 6 of the 16 samples detected c.130-131G mutation (Fig. 6b). In contrast, 11 of the 16 PQ-treated cells detect 0.024% to 0.73% of c.130-131G mutations (Fig. 6a). The G misrepair rate in the PQ-treated group was also higher than the control group (Additional file 1: Fig. S6b). Altogether, these data provide strong evidence that ROS promotes *MED12* c.130-131GG mutations in myometrial cells *in vitro*. This is a discovery of substantial value to the field as proof of concept showing hotspot mutations in *MED12* caused by inducers of ROS in the myometrium. Findings support *MED12* exon 2 mutations at c.130-131G can be induced by oxidative exposure *in vitro* and *in vivo*.

## Discussion

In this study, we addressed important gaps in knowledge regarding oxidative stress in the myometrium and *MED12* mutations in LM development. As proof of concept, we demonstrated that ROS could promote *MED12* mutations, at c.130-131 in exon 2, in myometrial cells, suggesting that mutation in this region could be an early, initiating event for LM. It is thought that the majority of LM result from transformations of a myocyte after a single genetic hit. Thus, there is high interest in the mutations found in *MED12* in more than 70% of uterine LM. We have shown here, a significantly higher rate of *MED12* mutations in uteri with a greater number of LM (Fig. 1). The positive correlation of *MED12* mutations with LM tumor numbers in the uterus has been reported previously [29, 30], and the reason for this may surround the increased oxidative stress found with high numbers of LM occur, shown in this study.

The uterus is considered to be an organ that is naturally associated with high oxidative stress. The myometrium of reproductive-age women undergoes cyclic contractions promoting local hypoxia [31] and is subject to inflammation during the menstrual cycle and menses [32]. Estrogen enhances ROS through redox cycling in mitochondria or proinflammatory cytokines [33]. To maintain homeostasis of the tissue, detoxifying mechanisms are triggered to offset the harmful effects of ROS, including the activity of MnSOD, followed by catalase, glutathione peroxidases, and the antioxidative pathway (HO-1). Recently, we and others demonstrated that LM have insufficient detoxifying mechanisms, including reduced MnSOD activity [12], altered AKT signaling [21], and altered ROS-induced miRNA response[34] and NADPH oxidase complex profile [13]. Whether the early *MED12* mutations are involved in driving these responses remains to be investigated.

ROS radical that induces most damage to DNA is hydroxyl radical. DNA damage plays a significant role in mutations, genetic instability, and epigenetic changes. Many kinds of oncogenes and tumor suppressor genes can suffer damage by oxidative stress causing mutations. The oxidized guanine (8-OHdG) is mutagenic, and many studies showed that levels of 8-OHdG are elevated in many different types of cancer. 8-OHdG can pair with both adenine and cytosine, but if the mismatch between adenine and guanine (A: G) is not repaired, there will be a transversion of regular pairs of adenine and thymidine (A: T), cytosine and guanine (C: G) that is the hallmark of oxidative mutagenesis [14, 18]. This mutation is commonly found in oncogenes and tumor suppressor genes. Detection of 8-OHdG misrepair induced by ROS has been tested at specific genes as well as globally [35] such as those found for *K-Ras* and *p53* [14, 15]. Therefore, 8-OHdG is used as a biomarker for the evaluation of oxidative stress. In this study, we found a high oxidative burden in myometrium with LM. This was demonstrated by a significantly increased 8-OHdG in the genomic DNA of myometrial cells. Oxidized genomic DNA in association with *MED12* mutations has never been investigated. *MED12* mutations in LM are mostly found in exon 2 and the intron 1 -exon 2 boundary, with a dominant hotspot at codon44 c.130G-131G (Fig. 1) [4]. *MED12* mutations occur in only a few tumor types, including LM and fibroadenoma of the breast [36] implicating selective mechanisms that are tissue-specific. We proposed in this study that increased ROS promotes 8-OHdG modifications which cause misrepair due to transversions of G nucleotide. If left unrepaired, 8-OHdG will base pair with dATP during replication to facilitate a stable G to T transversion.

A significantly high rate of 8-OHdG to T misrepair at codon44 c.130 in myometrial cells in vitro was detected (Figs. 4 and 5). It has been shown that the G to T transversion is the major targeted substitution mutation caused by the 8-OHdG base in mammalian cells [27]. A study by introducing 8-OHdG in target human lymphoblastoid TSCER122 cells resulted in high G:C to T:A transversions[27]. In nature occuring uterine leiomyoma, a G>A transition is dominant, accounting for >50% of c.130-c.131 mutations (Fig. 1). It is intriguing that c131G mutations from misrepair in vitro differ from those of native leiomyoma. One possible explanation is that c131G>A mutation is favorable for leiomyoma development regardless of frequency of mutation. Another plausible reason is that high ROS burden in myometrium can oxidized not only guanine in genomic DNA, but also can oxidize free GTPs which incorporate during DNA replication and then increase c131G>A transition [37].

Notably, a few control samples showed c.130G and c.131G alterations above baseline (Figs. 5c and 6b). These however would be rare and levels shown are at a much lower rate (<300/50k reads). Although we could not completely exclude the trace leiomyoma cell contamination, it was possible that myometrial cells were also subjected to *in vitro* culture conditions and therefore exposed to low levels of oxidative stress, and on occasion c130-131GG mutations could take place.

The overall frequency of mutations was not high and this is most likely attributed to the multiple repair systems that are in place, including BER and nucleotide excision repair (NER), that can repair oxidized nucleotides[38]. Specifically, OGG1 (8-hydroxyguanine DNA glycosylase) and MUTYH (MutY DNA Glycosylase) are specific BER enzymes that repair modified guanine 8-OHdG and are responsible for the integrity of ROS-induced DNA modification [38]. However, correction of 8-OHdG *in vivo* is not perfect, and a small fraction of misrepair and nucleotide transversions occur [27]. In this study, we found altered BER gene expression in myometrium and LM with increased 8-OHdG levels (Fig. 3), indicating the presence of single base repair responses in these cell types. This may be one explanation of why LM tumors are not ridden with mutations compared to other malignant tumors. It would be of interest to analyze other regions of the genome in our experimental model and compare them with naturally occurring mutations in LM.

The limitation of this study is that we only presented PQ-mediated *MED12* mutations. Although PQ is a reliable oxidative inducer, it remains unknown whether PQ can reproduce ROS environment similar to nature uterus. Our preliminary data (not shown) showed that other ROS inducers, such as KBrO_3_ and H_2_O_2_ can induce *MED12* mutations similar to PQ, but their reproducibility and remarkable genotoxic effects in global transcriptome make them less favorable in ROS-mediated mutation analysis. The transgenic models of SOD2 and NOX4 can be an excellent alternative in the future studies.

## Conclusion

In summary, this is the first study to demonstrate the association between uterine oxidative stress and *MED12* mutations arising through 8-OHdG misrepair. Further research is needed to understand how *MED12* mutation in myometrial cells promotes transformation into LM tumors.

## Supplementary Information

**Additional file 1**: **Figure S1**. *MED12 *mutation patterns and distribution of leiomyomas (LM). **Figure S2**. Immunostaining of ROS and DNA damage markers in tissues of different types. **Figure S3**. Validation of ROS and DNA damage, as well as the cell similarities of the myometrial cells treated with PQ and KBrO_3_. **Figure S4**. Pathway analysis and validation of mRNA expression in myometrial cells treated with ROS inducers. **Figure S5**. CRISPR/Cas9-mediated targeted replacement of c.130G with 8-oxodG and misrepair analysis in myometrial cells. **Figure S6**. Heatmap of deep sequencing and dot plot of duplex sequencing for cells treated with PQ

**Additional file 2: Table S1.** Case list and annotation. **Table S2.** Primers and oligonucleotide sequence information. **Table S3.** Antibody. **Table S4.** Deep sequencing and Duplex Sequencing data. **Table S5.** RNA sequencing data

## Data Availability

The datasets supporting the conclusions of this article are included within the article and Supplementary Information.

## Abbreviations

AP: apurinic or apyrimidinic
BER: base excision repair
Cas9: CRISPR associated protein 9
CRISPR: clustered regularly interspaced short palindromic repeats
DHE: dihydroethidium
DS: duplex sequencing
FFPE: formalin-fixed and paraffin embedded tissues
GSEA: Gene Set Enrichment Analysis
GATK4: genome Analysis Toolkit 4
KBrO_3_: potassium bromate
LM: leiomyoma
MED12: mediator complex subunit 12
NER: nucleotide excision repair
8-OHdG: 8-hydroxy-2′-deoxyguanosine
8-oxo-dG: 8-oxo-2'-deoxyguanosine
PQ: paraquat dichloride hydrate
ROS: reactive oxidative species
SOD^K68Ac^: detoxify superoxide by protein acetylation at lysine 68
SOD^K122Ac^: detoxify superoxide by protein acetylation at lysine 122
TATAM: tracing DNA adducts in targeted mutagenesis
