# Supplementary material for: Myometrial oxidative stress drives MED12 mutations in leiomyoma
Source: Cell Biosci. 2022 Jul 22;12:111. doi: 10.1186/s13578-022-00852-0 (PMC9308324; doi:10.1186/s13578-022-00852-0)
Supplement: Supplementary file 1 — Additional file 1: Figure S1. MED12 mutation patterns and distribution of leiomyomas (LM). Figure S2. Immunostaining of ROS and DNA damage markers in tissues of different types. Figure S3. Validation of ROS and DNA damage, as well as the cell similarities of the myometrial cells treated with PQ and KBrO3. Figure S4. Pathway analysis and validation of mRNA expression in myometrial cells treated with ROS inducers. Figure S5. CRISPR/Cas9-mediated targeted replacement of c.130G with 8-oxodG and misrepair analysis in myometrial cells. Figure S6. Heatmap of deep sequencing and dot plot of duplex sequencing for cells treated with PQ [file 13578_2022_852_MOESM1_ESM.pdf]

Figure S1

a

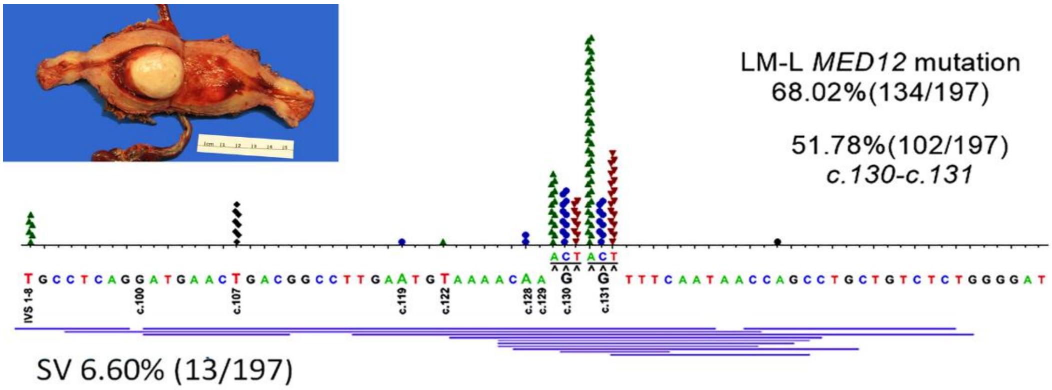

b

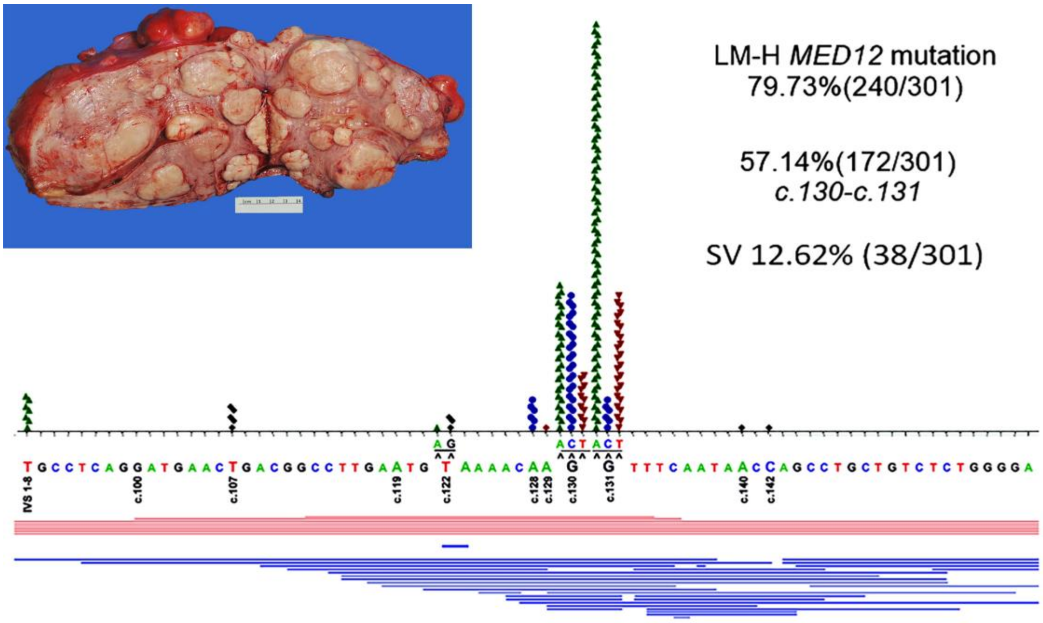

Supplementary Figure S1. ***MED12*** **mutation patterns and distribution of leiomyomas (LM).** (**a-b**) *MED12* mutation patterns and distribution of leiomyomas (LM) based on number of tumors/uterus. Insert photos show examples of gross appearance of uterine LM of each type.

Figure S2

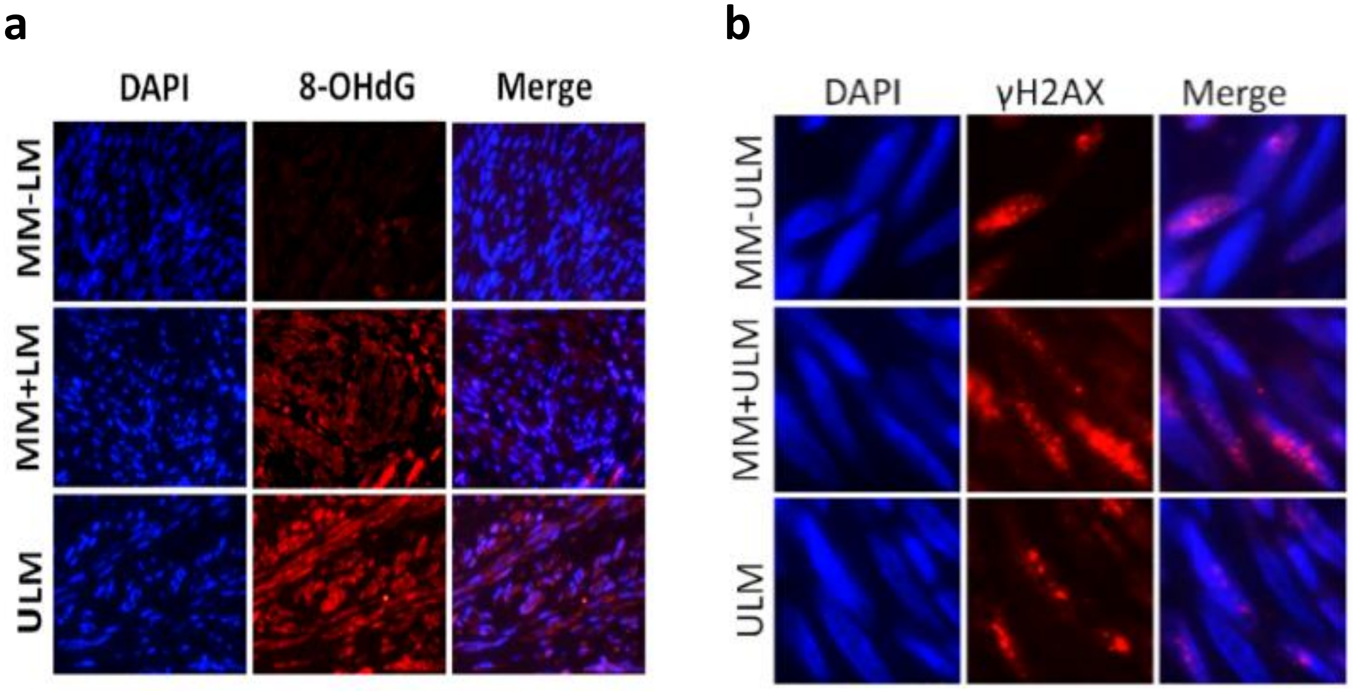

Supplementary Figure S2. Immunostaining of ROS and DNA damage markers in tissues of different types.

(a) Immunofluorescence staining of oxidized guanine (8-OHdG) in myometrium without leiomyoma (MM^{-LM}), myometrium with leiomyoma (MM^{+LM}), and leiomyoma (ULM). **(b)** γH2AX immunofluorescence staining in MM^{-LM}, MM^{+LM}, and LM. DAPI staining of DNA shown in blue.

Figure S3

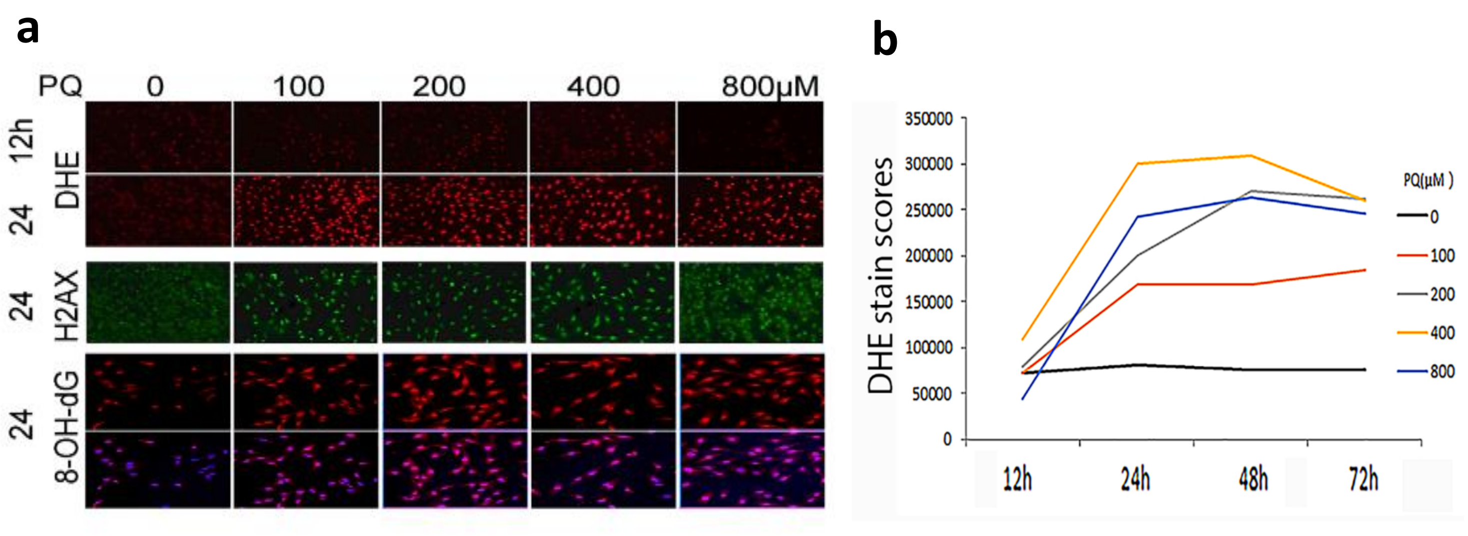

Supplementary Figure S3. Validation of ROS and DNA damage, as well as the cell similarities of the myometrial cells treated with PQ and $KBrO_3$.

(**a**) Dihydroethidium (DHE) staining to detect ROS level, immunofluorescence staining for 8-OHdG to detect oxidized DNA, and immunofluorescence staining of γH2AX to detect DNA damage in primary cultures of myometrial cells treated with different concentrations of PQ at different time points. (**b**) DHE stain scores in response to PQ treatment over time.

Figure S4

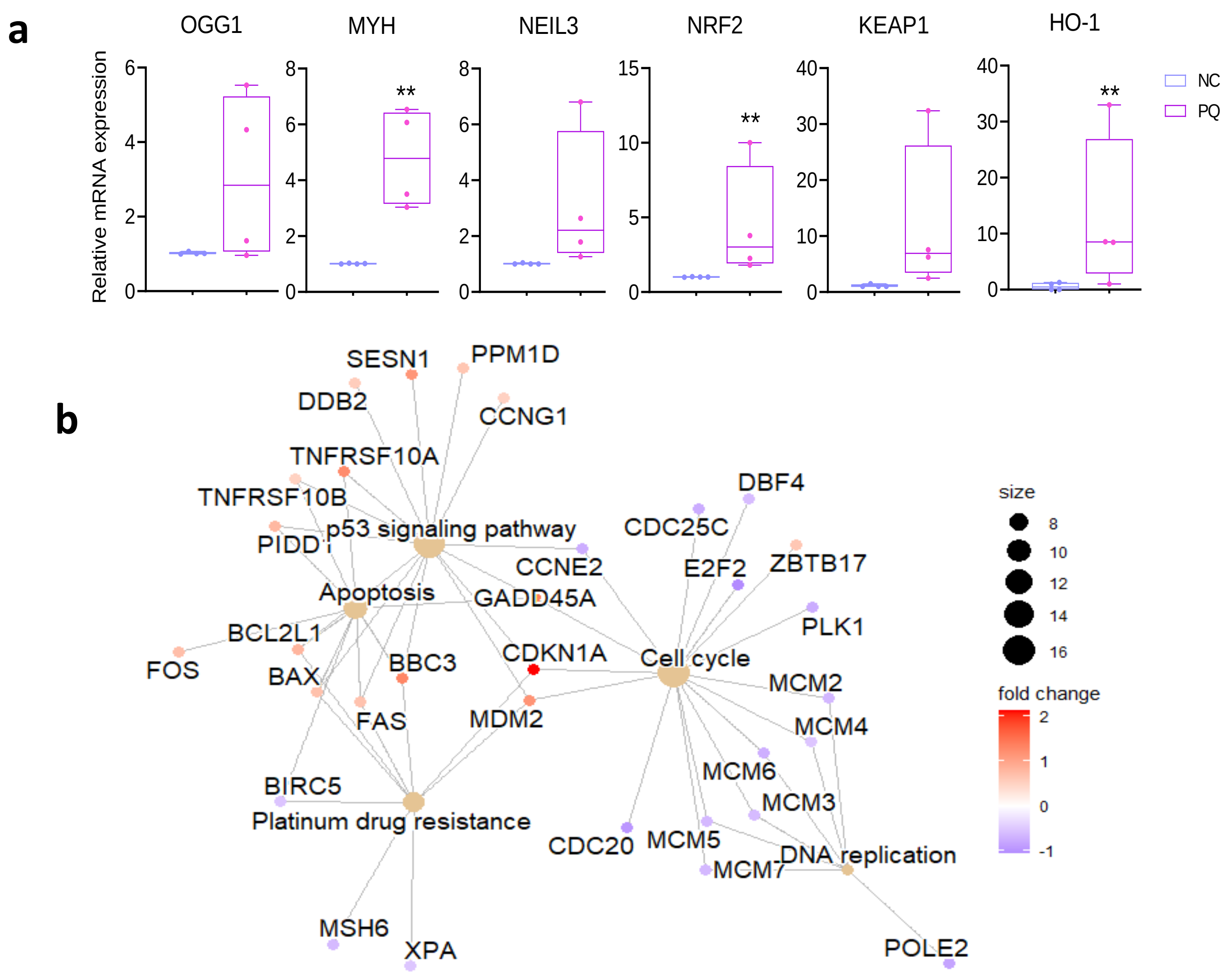

Supplementary Figure S4. Pathway analysis and validation of mRNA expression in myometrial cells treated with ROS inducers. (**a**) Real time RT-PCR analysis of the selected genes in 4 cases of myometrial samples treated with PQ. (**b**) Pathway connections and expression trends in cells treated with PQ. The relative gene expression level is ranked in low (purple) to high (red).

Figure S5

a

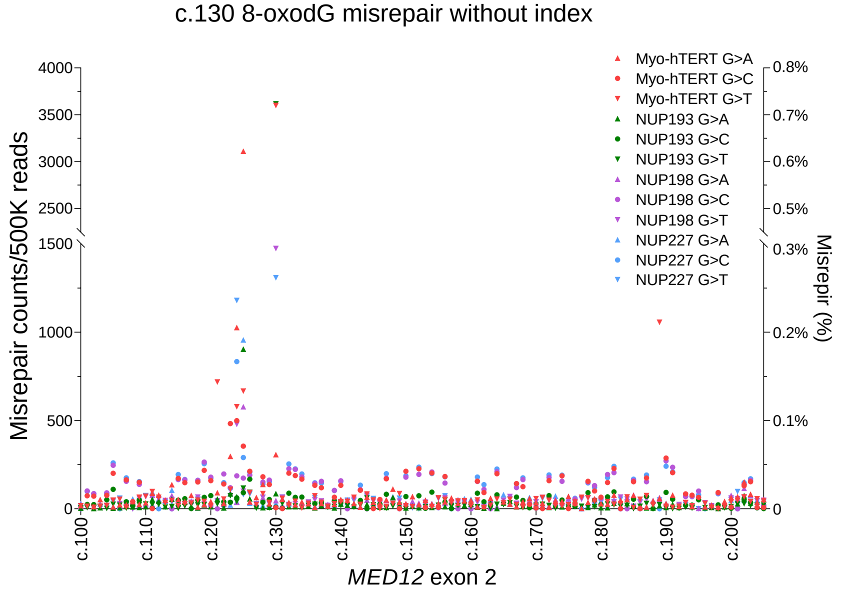

b

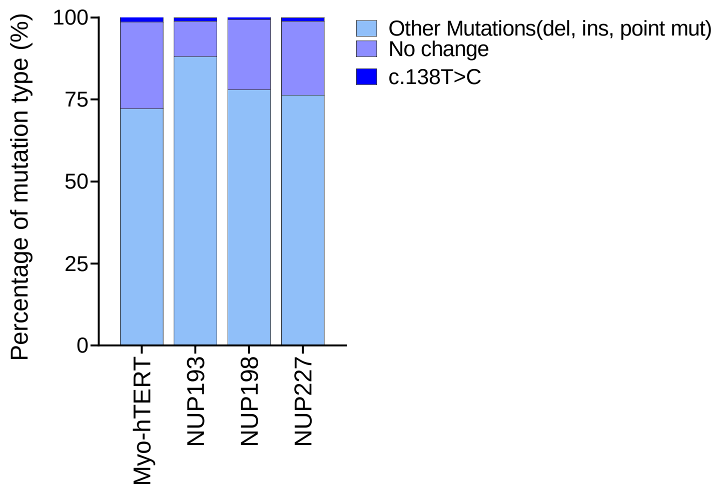

Supplementary Figure S5. CRISPR/Cas9-mediated targeted replacement of c.130G with 8-oxodG and misrepair analysis in myometrial cells. (a) Dot plot revealed the misrepair reads with CRISPR/Cas9 editing to c.130$^{8\text{-oxodG}}$ in *MED12* exon2 in high depth deep sequencing analysis (500k reads/sample). **(b)** Histobars showed the percentage of different mutation types with CRISPR/Cas9 editing in four myometrial samples.

Figure S6

a

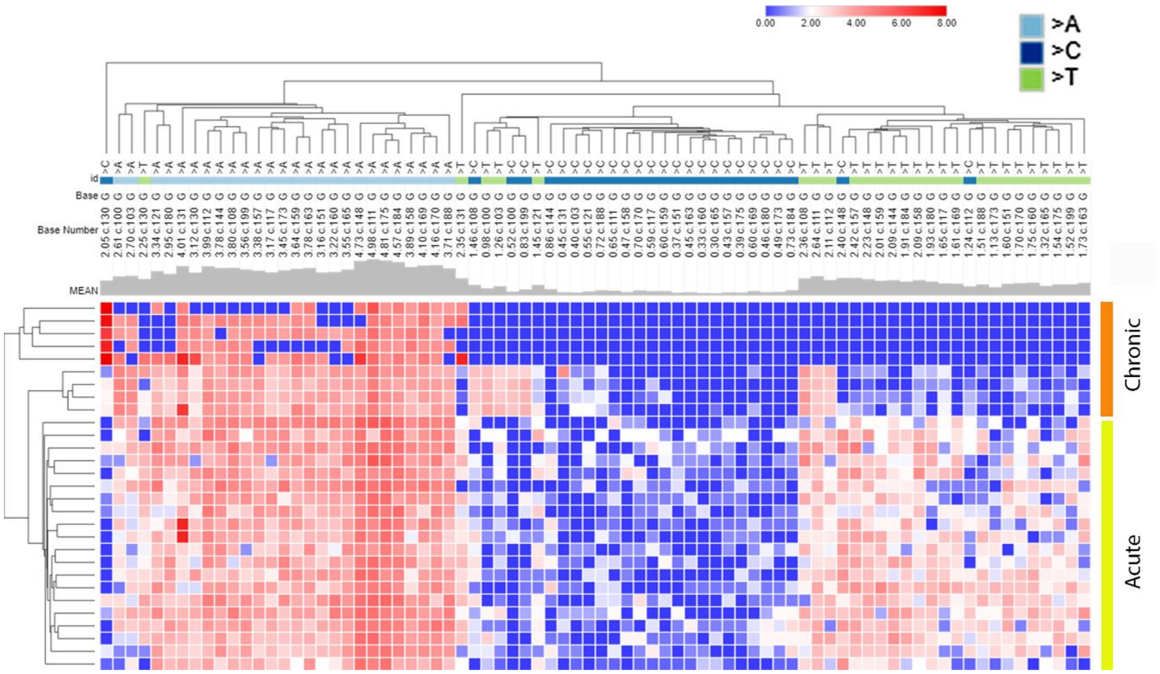

b

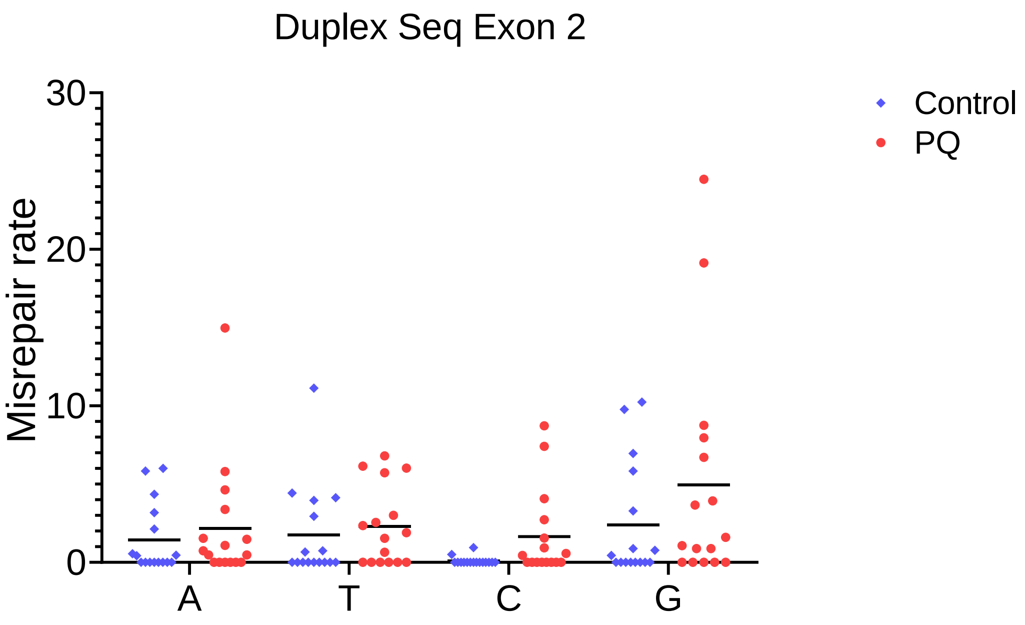

Supplementary Figure S6. Heatmap of deep sequencing and dot plot of duplex sequencing for cells treated with PQ. (**a**) Heatmap indicating the mutation patterns of G>A, G>C and G>T across exon 2 of *MED12* in myometrial cells treated with PQ, detected by deep sequencing. (**b**) The average misrepair rate of TCGA in *MED12* exon 2 in myometrial cells treated with control and PQ, detected by duplex sequencing analysis.
